# Dexamethasone–tamoxifen combination exerts synergistic therapeutic effects in tamoxifen-resistance breast cancer cells

**DOI:** 10.1042/BSR20240367

**Published:** 2024-07-05

**Authors:** Aliaa I. Gaballah, Aliaa A. Elsherbiny, Marwa Sharaky, Najat O. Hamed, Nahed A. Raslan, Abdullah Almilaibary, Reda Mohamed Abdrabbou Fayyad, Mona S. Ousman, Ahmed M.E. Hamdan, Sally A. Fahim

**Affiliations:** 1 School of Pharmacy, Newgiza University (NGU), Newgiza, km 22 Cairo-Alexandria Desert Road, Giza, P.O. Box 12577, Egypt; 2 Department of Biochemistry, School of Pharmacy, Newgiza University (NGU), Newgiza, km 22 Cairo-Alexandria Desert Road, Giza, P.O. Box 12577, Egypt; 3 Pharmacology Unit, Department of Cancer Biology, National Cancer Institute, Cairo University, Giza, Egypt; 4 Department of Pharmaceutical Sciences, College of Pharmacy, AlMaarefa University, P.O. Box 71666, Riyadh 11597, Saudi Arabia; 5 Department of Pharmacology and Toxicology, Faculty of Pharmacy (Girls), Al-Azhar University, Cairo 11651, Egypt; 6 Clinical Pharmacy Program, College of Health Sciences and Nursing, Al-Rayan Colleges, Medina 42541, Saudi Arabia; 7 Department of Family and Community Medicine, Faculty of Medicine, Al-Baha University, AlBaha, Saudi Arabia; 8 Department of Pharmacology, Faculty of Medicine, Al-Azhar University, Cairo, Egypt; 9 Department of Pharmacology, General Medicine Practice Program, Batterjee Medical College, Aseer 61961, Saudi Arabia; 10 Emergency Medical Services, College of Applied Sciences, AlMaarefa University, P.O. Box 71666, Riyadh 11597, Saudi Arabia; 11 Department of Pharmacy Practice, Faculty of Pharmacy, University of Tabuk, Tabuk 71491, Saudi Arabia

**Keywords:** breast cancer, dexamethasone, E2F3, SOX-2, synergistic effect, tamoxifen-resistance

## Abstract

Tamoxifen (TAM) is a key player in estrogen receptor-positive (ER+) breast cancer (BC); however, ∼30% of patients experience relapse and a lower survival rate due to TAM resistance. TAM resistance was related to the over expression of SOX-2 gene, which is regulated by the E2F3 transcription factor in the Wnt signaling pathway. It was suggested that SOX-2 overexpression was suppressed by dexamethasone (DEX), a glucocorticoid commonly prescribed to BC patients. The aim of the present study is to explore the effect of combining DEX and TAM on the inhibition of TAM-resistant LCC-2 cells (TAMR-1) through modulating the E2F3/SOX-2-mediated Wnt signaling pathway. The effect of the combination therapy on MCF-7 and TAMR-1 cell viability was assessed. Drug interactions were analyzed using CompuSyn and SynergyFinder softwares. Cell cycle distribution, apoptotic protein expression, gene expression levels of SOX-2 and E2F3, and cell migration were also assessed. Combining DEX with TAM led to synergistic inhibition of TAMR-1 cell proliferation and migration, induced apoptosis, reduced SOX-2 and E2F3 expression and was also associated with S and G2-M phase arrest. Therefore, combining DEX with TAM may present an effective therapeutic option to overcome TAM resistance, by targeting the E2F3/SOX-2/Wnt signaling pathway, in addition to its anti-inflammatory effect.

## Introduction

Breast cancer (BC) is the most widely diagnosed cancer globally, with about 2.3 million females diagnosed in 2020 [1]. It also accounted for over 685,000 female deaths, making it the second leading cause of cancer death worldwide [2,3]. Additionally, more than 3 million new cases and 1 million deaths are predicted to occur in 2040 [4]. Similarly, in Egypt, BC was the most diagnosed cancer and the second leading cause of cancer mortality in 2020 [5].

Estrogen is a major player in the development of BC and approximately 80% of cases are estrogen receptor positive (ER+) [6]. The carcinogenicity of estrogen can be attributed to three primary mechanisms: directly causing genotoxicity, inducing cellular proliferation, and promoting aneuploidy [7]. Due to the significant involvement of the ER in BC, endocrine therapy plays a critical role in treating the disease [8]. Three classes of endocrine therapy are mainly used: selective estrogen receptor modulators (SERMs), selective estrogen receptor down regulators, and aromatase inhibitors [9]. The most used therapeutic agent to treat ER+ BC is tamoxifen (TAM), a SERM. In the breast tissue, TAM exerts an antagonistic effect as it competes with estrogen for binding with the ER, displacing estrogen and inhibiting its pro-tumor functions [10]. TAM was reported to reduce the relapse and mortality of BC by approximately 40% and 30%, respectively. Despite being the first choice of therapy for ER+ BC, 20–30% of patients demonstrated TAM resistance, limiting its therapeutic efficacy [9]. TAM resistance leads to the relapse of many ER+ BC patients and reduces the survival rate to less than 20% [11,12]. The exact mechanism remains unclear; however, studies have demonstrated that protective autophagy, cell cycle regulators, and certain transcription factors are involved in the development of TAM resistance [13–15]

Sex determining region Y (SRY)-like box 2 (SOX-2) is a well-characterized transcription factor that was linked to the emergence of TAM resistance [16,17]. It belongs to the SOX gene family, which plays vital roles in cell development, proliferation, and differentiation, in addition to promoting cancer cell stemness and metastasis in different cancer types, such as breast, lung, and ovarian cancer [18,19]. It was also found to promote angiogenesis through up-regulation by VEGFA [20]. SOX2 was reported to promote breast cancer through targeting miR-181a-5p and miR-30e-5p/TUSC3 axis [18].

SOX-2 was suggested to be regulated by the E2F transcription factor 3 (E2F3), which is one of the E2F family of transcription factors which are key players in cell cycle regulation [19,21,22]. E2F3 interaction with SOX-2, altered its expression levels in TAM resistant cells, and its involvement in regulating pluripotency, differentiation of cancer stem cells (CSC), and the Wnt signaling pathway suggests that it might be involved in the emergence of TAM resistance [23]. Thus, E2F3/SOX-2-mediated Wnt signaling may present an attractive therapeutic target to overcome TAM resistance.

Corticosteroids have potent anti-inflammatory properties and are commonly used in the treatment of several cancer-related indications [24]. Glucocorticoids are a class of corticosteroids that are widely prescribed for cancer patients due to their anti-inflammatory, anti-allergy, and anti-edema effects [25]. Moreover, glucocorticoids showed various anticancer effects on different types of cancers including leukemias, lymphomas, and myelomas [25,26].

Dexamethasone (DEX) is the most commonly prescribed glucocorticoid that functions by binding to the glucocorticoid receptor (GR) [27]. It is used for the management of cancer-related pain due to its high potency, long duration of action, and minimal mineralocorticoid properties [28,29]. The effects of DEX vary depending on the subtype of BC. High expression of GR is associated with a poor prognosis in ER − BC and a good prognosis in ER + BC [30]. GR modulation decreases ER + BC proliferation and contributed to chemotherapy resistance [31,32]. While in ER − BC patients, the activation of GR is linked to the expression of genes associated with tumor survival and resistance to chemotherapy. These genes are involved in pathways that inhibit apoptosis and promote epithelial-ṇmesenchymal transition [33,34].

A study by Wang et al. [35] suggested that pretreatment with DEX resulted in reduced myelosuppression and enhanced antitumor activity of certain chemotherapeutic agents, such as gemcitabine and carboplatin. DEX was reported to reduces tumor incidence through reduction of inflammatory signaling required for SOX-2-driven tumorigenesis.

The antiproliferative effect of DEX and its mechanism were not previously demonstrated on resistant BC cells. Thus, exploring the mechanism of action of the most commonly used glucocorticoid, DEX, on TAM resistant BC cells may provide important insights regarding possible synergistic effect on resistant cells by interfering with E2F3/SOX-2/Wnt signaling.

## Results

### Combining DEX and TAM synergistically inhibits the proliferation of TAM-resistant LCC-2 cells (TAMR-1)

**Figure 1:**
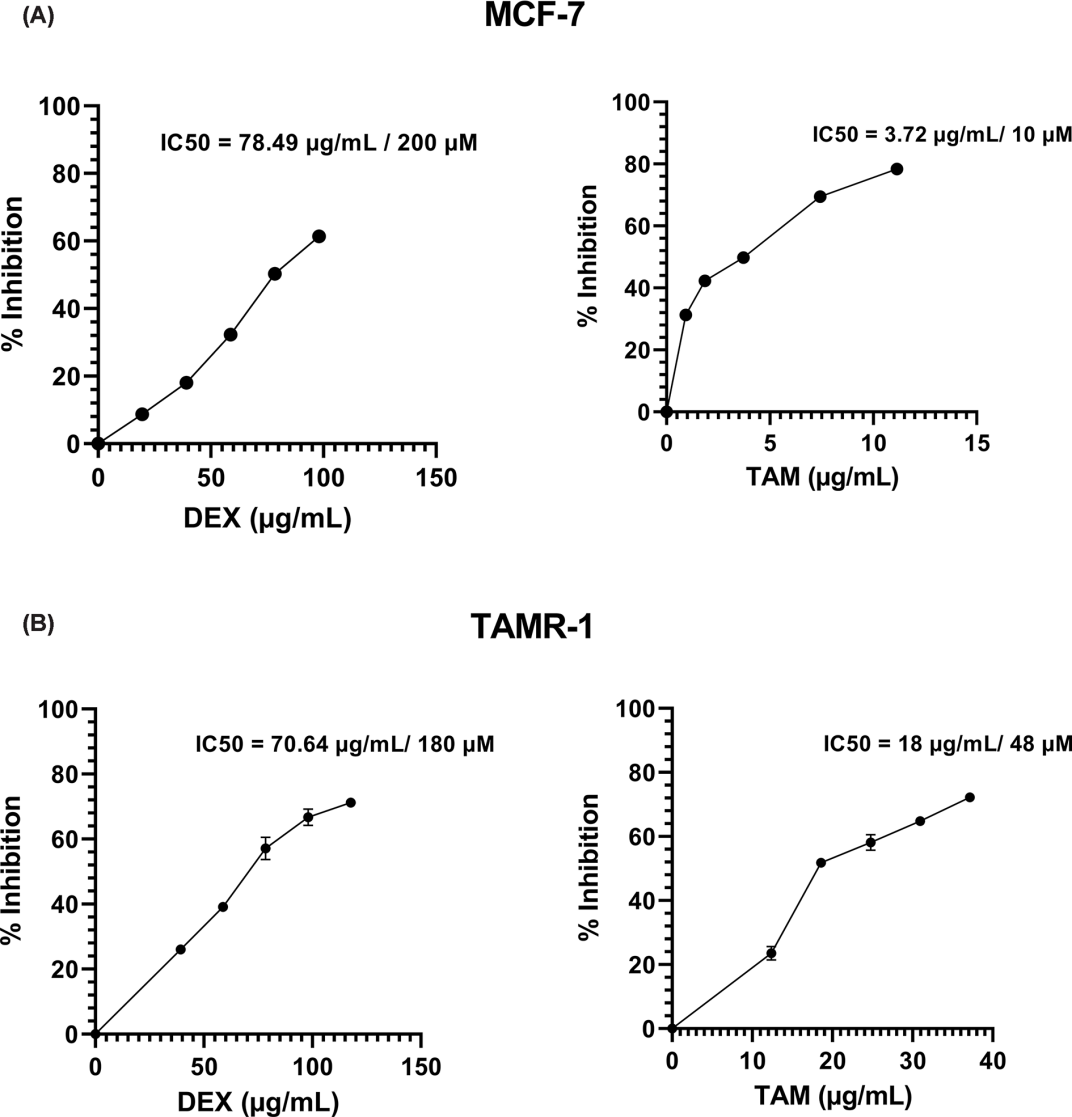
Anticancer activity of DEX and TAM on MCF-7 and TAMR-1 cells MCF-7 cells were seeded into 96-well plate and treated with 39.25–117.74 μg/ml of DEX and 0.93–11.15 μg/ml of TAM (**A**). TAMR-1 was treated with 39.25–117.74 μg/ml DEX and 12.38–37.15 μg/ml TAM (**B**). Cells were then incubated for 48 h and stained with SRB. The inhibitory effect of each drug was assessed through determining the percentage inhibition, and IC50 values were identified. Percentage inhibition = 100 - (100 × optical density (treated cells)/optical density [untreated cells]).

The effect of DEX and TAM monotherapy on MCF-7 and TAMR-1 cells was assessed through SRB cell viability assay. Both drugs were observed to have an inhibitory effect on both cell lines in a dose-dependent manner. Using the generated dose–response curves, IC50 values of DEX and TAM were determined. In MCF-7 cells, the IC50 values of DEX and TAM were 200 µM/78.49 µg/ml and 10 µM/3.72 µg/ml, respectively (Figure 1A). In TAMR-1 cells, the IC50 values of DEX and TAM were 180 µM/70.64 µg/ml and 48 µM/18 µg/ml, respectively (Figure 1B). It is obvious that TAMR-1 cells showed a 4.8-fold increase in IC50 value of TAM compared with MCF-7 cells.

In Vero normal cells, DEX showed a significantly lower cytotoxicity compared with TAMR-1 cells (*P*<0.0001), where the maximum dose (117.7 µg/ml) used inhibited normal cells viability by less than 25% compared with 71% in TAMR-1 cells (Figure 1C).

**Figure 2:**
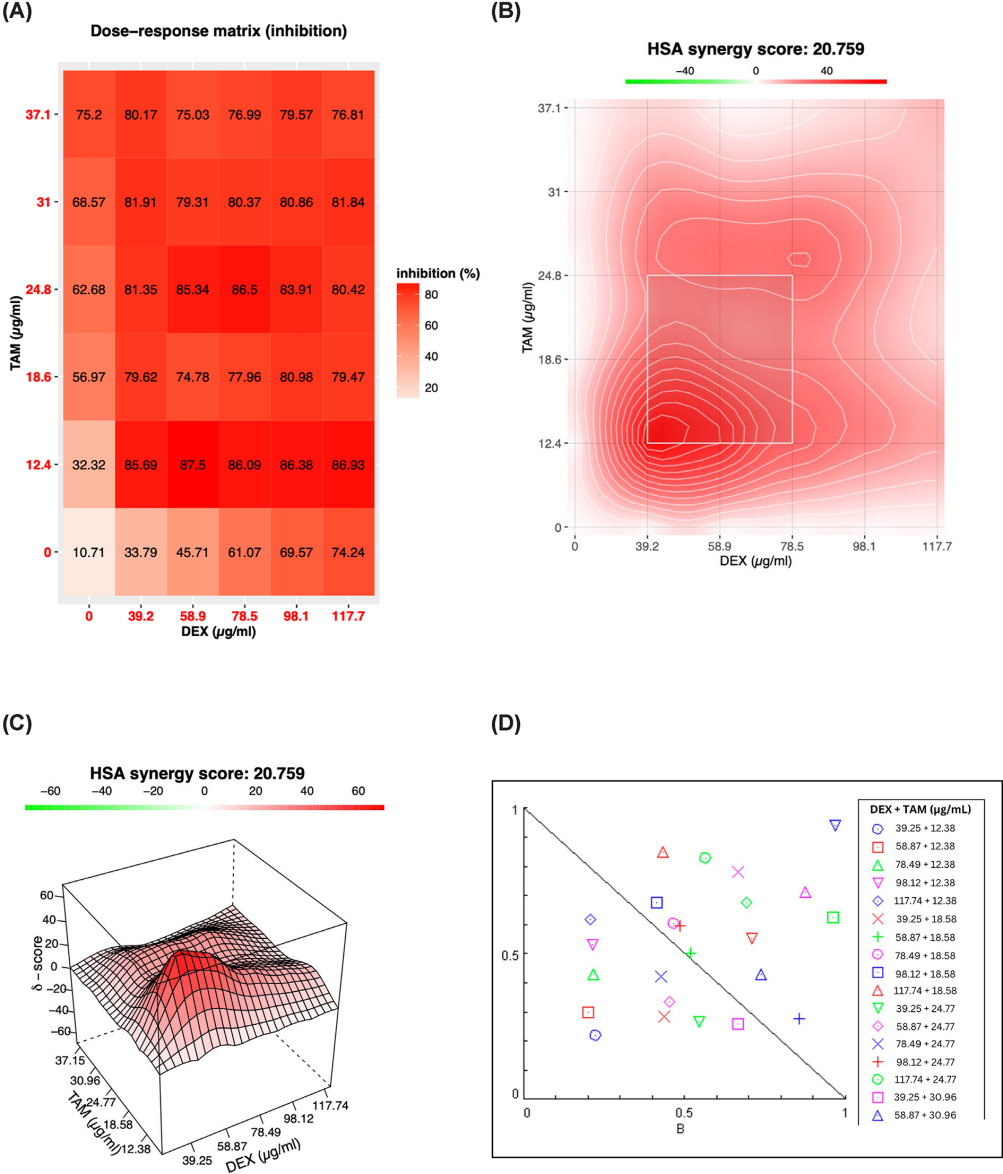
Inhibitory effect of DEX and TAM combination on TAMR-1 cells Percentage inhibition of TAMR-1 cells treated with increasing concentrations of DEX and TAM in a constant 3:1 ratio (**A**) for 48 h. The shade of red represents the amount of inhibition of TAMR-1 cells. (**B,C**) 2D and 3D synergism visualization showing the presence of synergy between DEX and TAM combination therapy. SynergyFinder 3.0 software was used to visualize the percentage inhibition of DEX and TAM combination therapy as a dose-response matrix, and the 2D and 3D visualization of synergy between DEX and TAM. The synergy score was calculated using the HSA reference model. (**D**) A Normalized Isobologram represents the fraction affected (Fa) and the CI values of different DEX and TAM combinations. CompuSyn software was used to confirm the presence of synergy between DEX and TAM through calculating the CI. CI < 1 suggests a synergistic effect, CI = 1 suggests an additive effect, and CI > 1 suggests an antagonistic effect.

The DEX and TAM combination regimen used in TAMR-1 cells was chosen according to their IC50 values in monotherapy. TAMR-1 cells were treated with a constant DEX to TAM ratio of 3:1, with DEX concentrations ranging from 39.25 to 117.74 μg/ml and TAM from 12.38 to 37.15 μg/ml, for 48 h. The SRB assay showed a decrease in cell viability in all combination-treated cells. Cells treated with 12.38 μg/ml of TAM showed a consistently high percentage of inhibition with all concentrations of DEX used, with an inhibitory effect of 85.7% when combined with 39.25 μg/ml of DEX. Combining 39.25 μg/ml of DEX with 12.38 μg/ml of TAM increased the percentage inhibition by 53.38% compared with TAM monotherapy (Figure 2A).

SynergyFinder 3.0 software was used to determine the type of interaction between DEX and TAM using the Highest Single Agent (HSA) reference model. 2D and 3D synergy heatmaps indicated that DEX and TAM exhibited a synergistic interaction through a wide range of concentrations with a synergy score of 20.76, indicating a 20.76% of response beyond expectation (Figure 2B,C). The HSA synergy score greater than 10, from 10 to -10, or less than -10, indicates synergism, additivity, or antagonism, respectively [36]. Moreover, CompuSyn software was used to confirm the presence of synergy between DEX and TAM by calculating the CI using the Chou-Talalay equation [37], as well as identify the effect of combination therapy on decreasing the dose of TAM using the DRI. Combining 39.25 μg/ml of DEX and 12.38 μg/ml of TAM showed the lowest CI of 0.44 and a DRI of TAM equal to 4.47 (Figure 2D and Supplementary Table S1). Compared with monotherapy, this combination decreased the IC50 of TAM from 18 to 8 μg/ml in TAMR-1 cells.

The IC50 of TAM in TAMR-1 cells decreased from 18 to 8 μg/ml when combined with DEX. Based on the calculated synergy score, CI, and DRI, the combination regimen chosen for the following studies includes 39.25 μg/ml of DEX and 12.38 μg/ml of TAM.

### Combining DEX with TAM induces apoptosis in TAMR-1 cells

**Figure 3:**
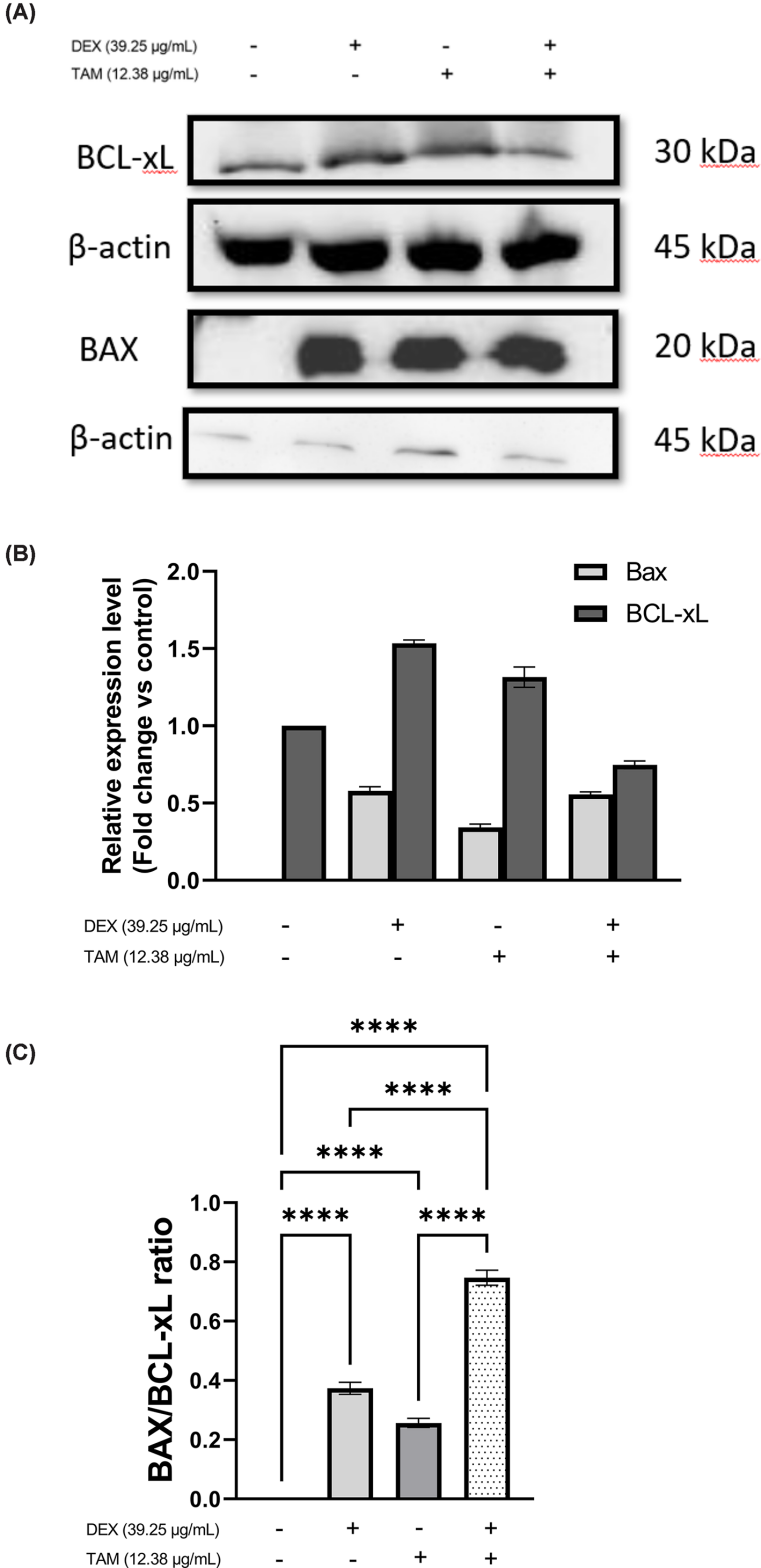
Western blotting assay of TAMR-1 cells Western blotting assay of TAMR-1 cells treated with 39.25 μg/ml DEX, 12.38 μg/ml TAM, or their combination for 48 h. (**A**) Western blotting images of Bax and BCL-xL expression levels in TAMR-1 cells. (**B**) Bar graph comparing the expression levels of Bax and BCL-xL. Data were normalized against β-actin and expressed as a fold change compared with control. (**C**) Bar graph representing Bax to BCL-xL ratio. Data is presented as mean ± SD. Statistical analysis was performed using one-way analysis of variance (ANOVA) followed by a Tukey’s post-hoc test. **** Significant at *P*<0.0001.

To investigate whether combining DEX with TAM induces apoptosis in TAMR-1 cells, expression levels of the pro-apoptotic protein Bax and the anti-apoptotic protein BCL-xL were measured using a Western blotting assay (Figure 3A). Untreated TAMR-1 cells represented controls. Combining DEX and TAM resulted in increased expression level of Bax compared with the control group and TAM treated cells, while decreased expression of BCL-xL as compared with both monotherapies and the control (Figure 3B). Moreover, the combination significantly increased the Bax/Bcl-xL ratio compared with each drug alone and the control (*P*<0.0001, Figure 3C).

### DEX and TAM combination therapy induces S phase arrest

**Figure 4:**
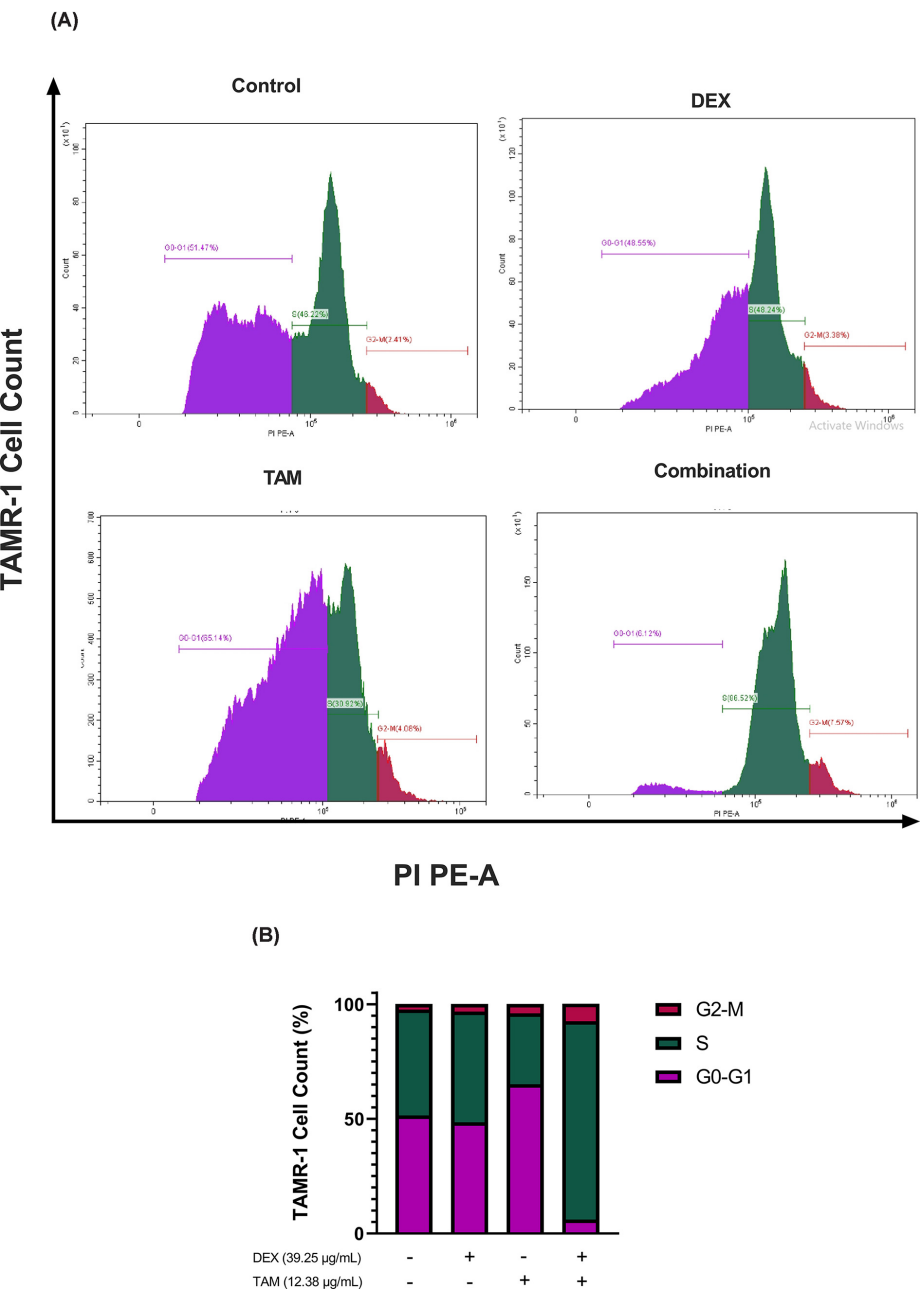
Cell cycle distribution of TAMR-1 cells Cell cycle distribution of TAMR-1 cells exposed to 39.25 DEX, 12.38 TAM, or their combination for 48 h. (**A**) Flow cytometry results showing percentage TAMR-1 cells arrested at each cell cycle phase (G0-G1, purple; S, green; G2-M, red). (**B**) Representative bar graph comparing percentage of cell cycle distribution in control and treated cells.

To confirm whether the inhibitory effect of DEX, TAM, or their combination is linked to cell cycle arrest, the cell cycle distribution of TAMR-1 cells was analyzed using a flow cytometer after treatment for 48 h. Cell treated with DEX and TAM monotherapy resulted in increased arrest at the G2-M phase (DEX: 3.38%, TAM: 4.08%) compared with the control (2.41%). Furthermore, combination treatment resulted in increased arrest at the S phase (86.52%), as well as the G2-M phase (7.57%), compared with the control (S: 46.22%, G2-M: 2.41%, Figure 4).

### Combining DEX and TAM significantly inhibits TAMR-1 cell migration

**Figure 5:**
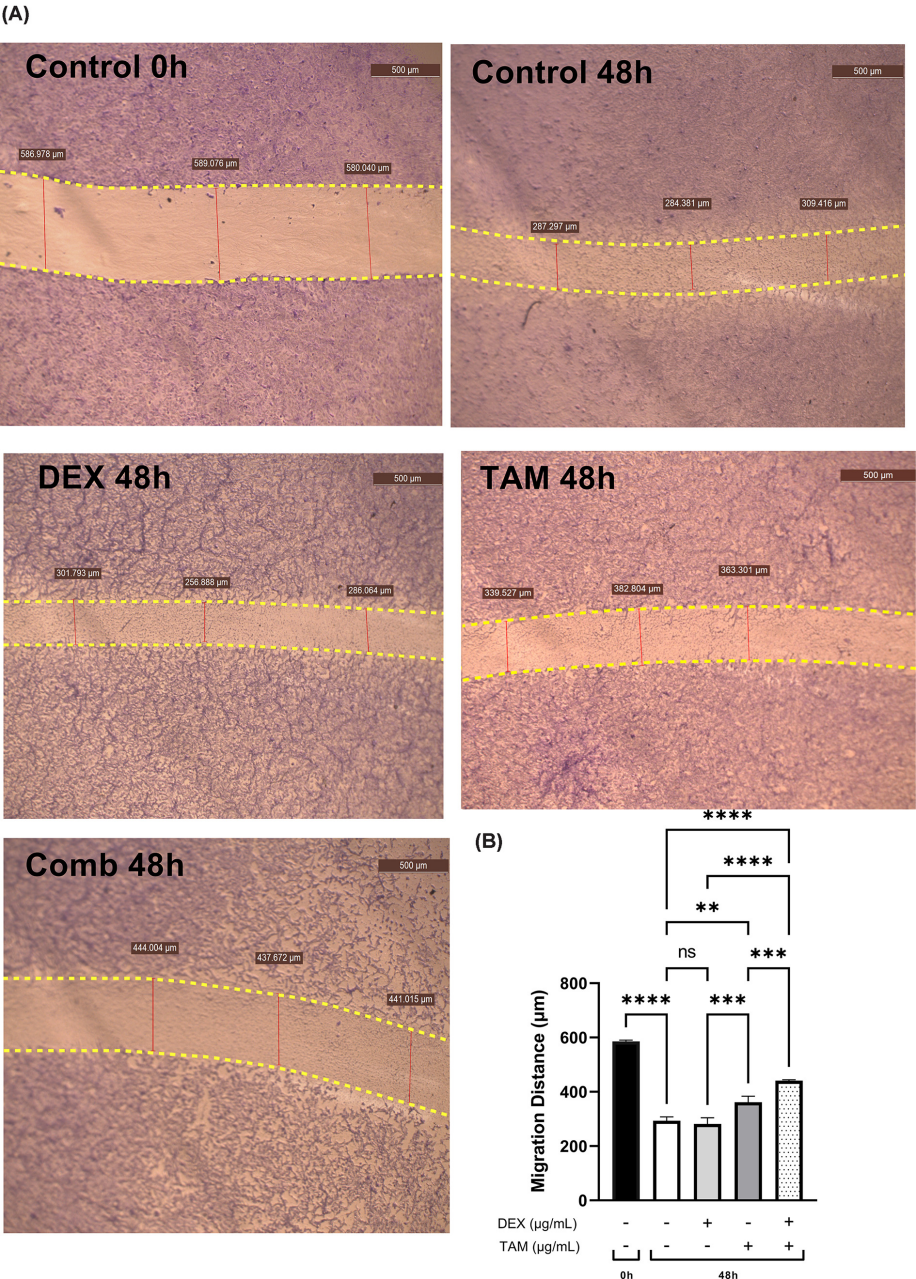
Wound healing assay of TAMR-1 cells Wound healing assay of TAMR-1 cells treated with 39.25 μg/ml DEX, 12.38 μg/ml TAM, or their combination for 48 h (**A**). Crystal violet was used as a staining agent. Representative bar graph showing the quantitative analysis of TAMR-1 cell migration presented as mean ± SD (**B**). Statistical analysis was performed using one-way analysis of variance (ANOVA) followed by a Tukey’s post-hoc test. **** Significant at *P*<0.0001, ***P<0.001, **P<0.01, ns = no significance.

The migratory ability of TAMR-1 cells after treatment with DEX, TAM, or their combination for 48 h was observed using a wound healing assay. Untreated TAMR-1 cells at 0 and 48 h represented controls (Figure 5A). While DEX monotherapy had no significant effect on cell migration (*P*>0.05), TAM monotherapy significantly inhibited TAMR-1 cell migration (*P*<0.01) when compared with the 48-h control. Furthermore, combining 39.25 μg/ml DEX and 12.38 μg/ml TAM led to the most significant delay of TAMR-1 cell migration (*P*<0.0001), as compared with monotherapy and the control (Figure 5B).

### DEX and TAM combination therapy significantly reduces expression of SOX-2 and E2F3 in TAMR-1 cells

**Figure 6:**
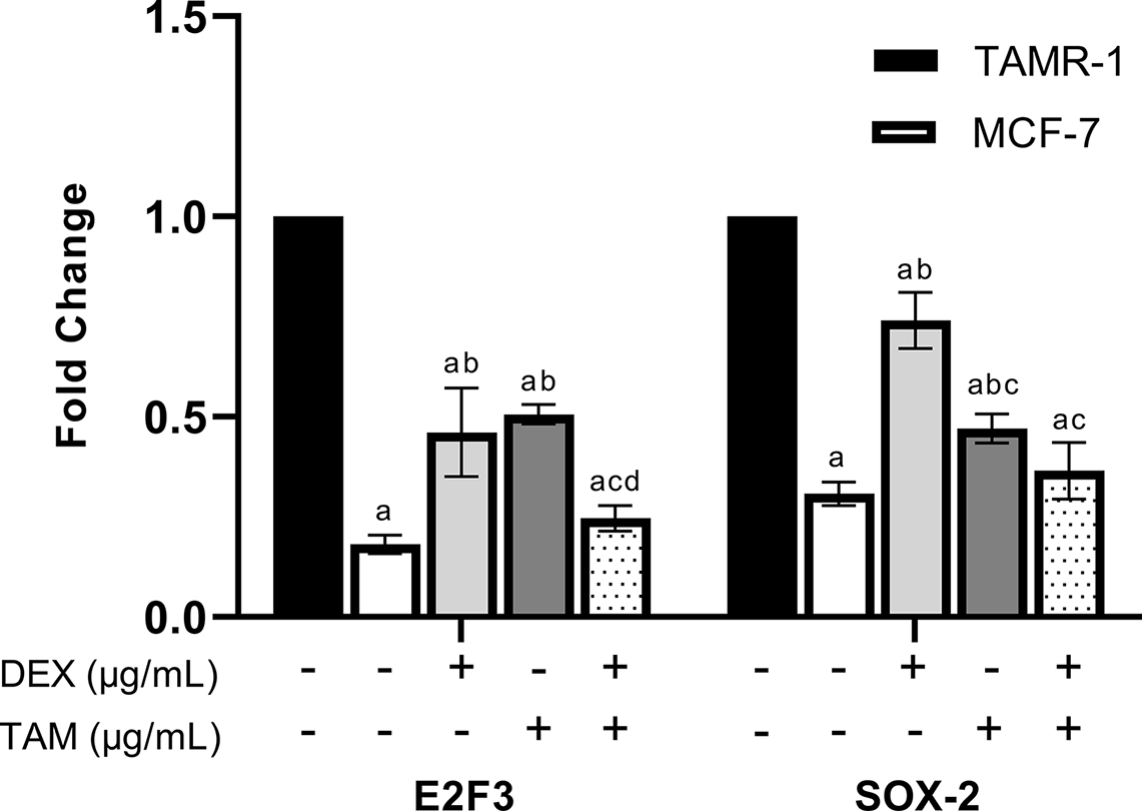
RT-qPCR analysis of SOX-2 and E2F3 mRNA expression in TAMR-1 cells RT-qPCR analysis of SOX-2 and E2F3 mRNA expression in TAMR-1 cells treated with 39.25 μg/ml DEX, 12.38 μg/ml TAM, or their combination for 48 h. Quantitative PCR results were normalized against GAPDH and expressed as a fold change. Data are acquired from three independent experiments and are presented as mean ± SD. Statistical analysis was performed using two-way analysis of variance (ANOVA) followed by a Tukey’s post-hoc test. (a, b, c, and d represent significance from TAMR-1, MCF-7, DEX, and TAM, respectively, at *P*<0.01).

To assess the effect of DEX on the expression of SOX-2 and E2F3 and their association with TAM resistance, RT-qPCR analysis was performed for MCF-7 and TAMR-1 cells treated with 39.25 μg/ml DEX, 12.38 μg/ml TAM, or their combination for 48 h. Data were normalized against TAMR-1 control. The results suggest that SOX-2 and E2F3 levels were significantly higher in TAMR-1 cells than in MCF-7 cells (*P*<0.0001). Monotherapy with either DEX, TAM, or their combination significantly reduced SOX-2 and E2F3 expression when compared with the TAMR-1 control at *P*<0.0001. Furthermore, combination therapy reduced SOX-2 and E2F3 expression as compared with DEX and TAM monotherapy. Interestingly, there was no significant difference between TAMR-1 cells treated with combination therapy and MCF-7 cells (Figure 6).

## Discussion

BC presents a global health concern [2,3]. Despite TAM is the first choice of therapy, the efficacy of TAM is restricted by the development of TAM resistance [9]. Previous studies suggested that one of the possible TAM resistance mechanisms involves the overexpression of the transcription factor SOX-2 [16,17].

DEX, a glucocorticoid commonly used in the treatment of BC-associated symptoms was previously suggested to reduce the activity of SOX-2 [38,39]. DEX was also previously found to enhance the cytotoxic effects of certain chemotherapeutic agents and suppress tumor growth and metastasis [40,41]. Based on these previous studies, the present study aimed to demonstrate the cytotoxic effect of DEX, TAM, or their combination on both MCF-7 and TAMR-1 BC cell lines by targeting the E2F3/SOX-2/Wnt signaling pathway.

SOX-2 was reported to play a strong role in promoting tumor progression in different cancer types. Its overexpression has been associated with increased proliferation, resistance to apoptosis, altered autophagy, and cancer stem cell (CSC) maintenance [21,42]. Zhu et al. [43] suggested that SOX-2 drives chemoresistance through β-catenin-mediated autophagy signaling. Moreover, Loh et al. [44] reported that the Wnt pathway was reported to be linked to TAM resistance in BC cells. On a transcriptional level, SOX-2 is up-regulated by E2F3, which is a cell cycle regulator found to be involved in progression of BC [45]. Analysis of SOX-2 and E2F3 gene expression was performed to assess the mechanisms underlying the inhibitory effect of DEX in TAMR-1 cells. The higher exxpression of SOX-2 in TAMR-1 cells in our study correlate with previous findings establishing that SOX-2 is overexpressed in resistant MCF-7 cells and silencing its expression increases sensitivity to TAM [46]. While E2F3 expression in TAMR-1 cells was not previously determined, previous studies reported that E2F3 expression was elevated in paclitaxel-resistant MCF-7 cells, as well as different resistant cancer types, including ovarian and lung cancer cells [47–49]. Treatment with either TAM or DEX or their combination lead to a significant decrease in SOX-2 and E2F expression to the levels of nonresistant cells. Similarly, a previous study reported that combination treatment leads to increased chemosensitivity of cancer cells through the down-regulation of SOX-2 [50]. A possible mechanism underlying the down-regulation of SOX-2 by DEX may involve inhibition of the Wnt/β-catenin pathway [51]. Furthermore, E2F3 was also found to activate the Wnt pathway through a mechanism involving the induction of MEX3A, leading to the suppression of the pro-differentiation transcription factor KLF4 [52]. Previous findings suggested that an excess of glucocorticoids interrupted the Wnt pathway through activation of Wnt-inhibitors, including Dickkopf-1 (Dkk-1), which was reported to be a tumor suppressor in BC as its inhibition leads to increased BC cell migration and invasion [53].

Our study has found that DEX treatment led to reduced cell viability in MCF-7 and TAMR-1 cell lines, with approximately equal IC50 values. This correlates to previous studies which demonstrated that DEX had pro-apoptotic effects on various cancer types such as leukemias, myelomas, and lymphomas [25,26]. Interestingly, DEX showed a significantly lower cytotoxicity on normal Vero cells compared with TAMR-1 cells. A previous study reported that DEX has demonstrated potential in mitigating the long-term effects of cytotoxic chemotherapy on normal tissues [54]. However, TAM treatment showed different IC50 values on MCF-7 and TAMR-1 cell lines of 3.72 and 18 μg/ml, respectively. The higher IC50 value of TAM on TAMR-1 cells indicates reduced sensitivity of the TAMR-1 cells in comparison to MCF-7 cells. This was also supported by previous studies showing similar disparity between TAM IC50 values in normal MCF-7 and resistant BC cell lines [55], with one study demonstrating a TAM IC50 value of 4.55 μg/ml on MCF-7 cells and 17.54 μg/ml on TAMR-1 cells [56]. Combining DEX and TAM resulted in increased inhibition of TAMR-1 cells when compared with TAM monotherapy. This corresponds with previous studies showing that DEX pretreatment enhances the antitumor activity of certain chemotherapeutic agents, such as carboplatin, gemcitabine, and adriamycin, by enhancing tumor uptake in various types of cancer, including BC [35,40]. It also correlates with previous findings suggesting that the administration of TAM in combination with other anti-inflammatory agents results in an increased inhibitory effect of TAM in both MCF-7 and TAMR-1 cells [57,58]. Furthermore, the IC50 of TAM in TAMR-1 cells decreased from 18 to 8 μg/ml when combined with DEX. This correlates with previous findings suggesting that combining DEX with anticancer agents, such as doxorubicin in lymphoma, gemcitabine in BC, and paclitaxel in BC results in an increased inhibitory effect with a decrease in paclitaxel IC50 from 19.3 to 5.22 μg/ml [59,60].

Interaction analysis using SynergyFinder 3.0 and CompuSyn software established the presence of synergy between DEX and TAM in TAMR-1 cells as indicated by the synergy score, CI, and DRI values [36]. This model assumes that combination treatment results in an expected effect equivalent to the higher effect of the individual drug at the same dose, supporting that the combinatorial treatment results in additional effects to those achieved by monotherapy [61].

Apoptosis is an important mechanism associated with the cytotoxicity of chemotherapeutic agents involving an intrinsic and an extrinsic pathway [62,63]. The BCL-2 protein family, which includes the proapoptotic protein Bax and the antiapoptotic protein BCL-xL, regulates apoptosis on an intrinsic level [64]. TAM resistance has previously been linked with apoptosis induced by the decreased levels of pro-apoptotic proteins including Bax [65]. The present study shows that Bax was not expressed in TAMR-1 control cells. Previous published data reported similar results in cancer and resistant cells [66–68]. Romero et al. [66] reported that the BCL-2 family protein; tBID might be able to directly induce apoptosis independently of BAX and BAK. Combining DEX and TAM increased expression of Bax and reduced expression of the BCL-xL. In MCF-7 cells, BCL-xL was found to be ten times more efficient than Bcl-2 in preventing apoptosis [69]. Previous findings suggested that the down-regulation of SOX-2 significantly induces apoptosis in various cancer types, including BC [70–72]. Moreover, SOX-2 was also found to induce resistance by increasing anti-apoptotic proteins like Bcl-2 and decreasing pro-apoptotic proteins like PUMA/BBC3 in ovarian cancer cells [73].

Cell cycle distribution of TAMR-1 cells was assessed to determine the mechanisms underlying the inhibitory effects of DEX and TAM treatment. The present study showed that DEX and TAM monotherapy led to arrest in the S phase of TAMR-1 cells. These findings correlate with previous studies showing that TAMR-1 cells treated with TAM were accumulated in the S phase [74]. Furthermore, in the current study, combination treatment was found to increase S and G2-M phase arrest. On the other hand, Cheng et al. [58] reported that combining TAM with another anti-inflammatory such as aspirin led to G0-G1 phase arrest in TAMR-1 cells. Although the effect of DEX on the cell cycle distribution of TAMR-1 cells was not previously explored, its monotherapy and combinatorial effect on other types of resistant cancers, such as lenalidomide-resistant multiple myeloma, was found to induce cell cycle arrest in the G0-G1 phase [75]. Furthermore, study by Liu et al. [18] reported that SOX-2 expression in BC cells increases levels of cell cycle proteins such as cyclin D1 as well as cyclin-dependent kinase 4 and 6, which were reported to be associated with the transition between G1 and S phases [76]. Another study by Feng et al. [77] reported that E2F3 overexpression promoted tumor progression through interfering with the cell cycle, as its knockdown led to increased G0-G1 phase arrest.

The present study suggests that migratory inhibition effect that results from combining DEX and TAM. Previous studies have found that TAM therapy inhibits migration in MCF-7 cells but not in the resistant TAMR-1 cells [78]. Furthermore, it was reported that combination treatment leads to a more significant delay in cancer cell migration when compared with TAM monotherapy [79,80]. DEX monotherapy and combination with other anticancer agents such as doxorubicin and docetaxel results in a significant inhibitory effect on BC cell migration [81–83]. However, Pang et al. [41] reported that while lower doses of DEX inhibit migration, higher doses (above 50 µg/kg) may promote BC progression. Furthermore, SOX-2 expression was found to be inversely correlated with expression of TUSC3 protein, which was reported to suppress BC cell proliferation and migration [18]. Moreover, SOX-2 was found to promote EMT through the Wnt/β-catenin signaling pathway, which induces the cell migration in BC [84]. Previous studies have also reported that SOX-2 and E2F3 may promote the migration of cancer cells [85–87]. These results indicate that combining DEX with TAM may suppress the proliferation and migration of TAMR-1 cells through a mechanism involving E2F3 and SOX-2 down-regulation.

DEX is moderate inducer to CYP3A4 so the metabolism of TAM can be increased when combined with DEX [88]. Endoxifen, an active metabolite of TAM, is produced through N-demethylation mediated by CYP3A4 and hydroxylation mediated by CYP2D6. When paroxetine is administered concurrently with TAM, it leads to a reduction in the plasma concentration of endoxifen [89]. Moreover, rifampin reduced the plasma concentrations of TAM by enhancing CYP3A4 [90]. On the other hand, combining TAM with DEX was beneficial in attenuating DEX-induced osteoperosis and osteopenia, and sensitizing glucocorticoid (GC)-resistant cell line Jurkat to DEX treatment [91,92]. More studies are needed to fully reveal the effect of DEX on TAM metabolism.

## Conclusions

SOX-2 and E2F3 overexpression could possibly contribute to TAM resistance. Combining DEX with TAM was found to synergistically inhibit the growth and migration of TAMR-1 cells. induce apoptosis, and lead to cellular arrest at the S and G2/M phases of the cell cycle. Moreover, the combination led to down-regulation of E2F3 and SOX-2 in TAMR-1 cells in levels as low as those of MCF-7 cells. Overall, our data suggest that combining DEX with TAM may present an effective therapeutic option to counteract TAM resistance, and the E2F3/SOX-2/Wnt signaling pathway may be a promising target for decreasing TAM resistance. Further research is recommended to investigate the downstream effects of E2F3/SOX-2/Wnt signaling pathway inhibition to gain a more comprehensive understanding of the molecular mechanisms underlying the observed effects. In addition, exploring the efficacy of DEX in other types of resistant cancer cells is also recommended. Moreover, clinical studies were also recommended to detect whether this pathway can be used as biomarker for the combination treatment and to evaluate the safety, efficacy, and optimal dosage of this combined therapy.

## Materials and methods

### Drugs and chemicals

DEX was supplied by EVA Pharma (Cairo, Egypt) and was stored at 2°C in an air-tight container. TAM was obtained from Amriya Pharmaceutical Industries (Alexandria, Egypt) and kept in storage at room temperature. Before use, TAM and DEX were dissolved in DMSO and then diluted with RPMI-1640 medium.

### Cell lines

The human BC MCF-7, LCC-2 TAM resistant BC (TAMR-1), and the normal Vero cell lines were provided by the American Cell Culture Collection (Minnesota, U.S.A.), and preserved in RPMI-1640 medium containing 10% fetal bovine serum (FBS) and 1% penicillin/streptomycin while incubated at 37°C, 5% CO_2_, and 95% humidity.

### Cell cultures

In 75-cm^2^ flasks, cancer cells were cultured in a monolayer in the presence of RPMI-1640 medium containing 10% FBS and antibiotics. Cells were subcultured in different flasks once 70% confluency was reached. Before use, the cultured cells adhered to cell culture flasks were dissociated with 0.5 ml of trypsin enzyme and incubated for 30 s at 37°C.

### SRB cell viability assay

In 96-well microtiter plates, cells were seeded at a concentration of 5000 cells per well and were left to adhere for 24 h before addition of the drugs. The cells were treated with varying concentrations of each drug as monotherapy. Initial screening was performed to determine the concentrations to be used. Based on the initial screening, both MCF-7 and TAMR-1 cell lines were treated with various concentrations of DEX (100–300 μM/39.25–117.74 μg/ml). Moreover, MCF-7 and TAMR-1 cells were treated with 2.5–30 μM (0.93–11.15 μg/ml) and 20–100 μM (12.38–37.15 μg/ml) of TAM, respectively. The plates were incubated for 48 h at 37°C and the optical density (OD) of each well was measured using an ELISA microplate reader (Sunrise, Tecan, Germany) at 570 nm. Cytotoxicity of DEX and TAM was determined through the sulforhodamine-B (SRB) method [93]. IC50 values were calculated using dose–response curves generated through Graphpad Prism 8.2.1.

According to the IC50, TAMR-1 cells were treated with a combination of DEX and TAM in a constant ratio of 3:1. The doses used in the combination regimen included the IC50 and doses higher and lower than the IC50 of monotherapy.

### Synergistic effect analysis

Synergistic analysis was performed using CompuSyn [94] and SynergyFinder 3.0 [95]. CompuSyn software was used to determine the combination index (CI) and dose reduction index (DRI). Using the CI, the type of drug interaction between DEX and TAM was determined, where CI < 1 suggests a synergistic effect, CI = 1 suggests an additive effect, and CI > 1 suggests an antagonistic effect. The DRI was used to determine how many folds the dose of TAM can be reduced in combination therapy as compared with monotherapy, where a DRI < 1 indicates negative dose reduction, DRI = 1 indicates no dose reduction, and DRI > 1 indicates favorable dose reduction. SynergyFinder 3.0 software was used to determine a synergy score, using the Highest Single Agent (HSA) reference model [96,97].

### Western blot assay

Proteins were extracted from different cells by adding 10 mM Tris-HCl, 100 mM NaCl, 0.5% Triton X-100, pH 7.6 with EDTA-free Protease Inhibitor. A Pierce™ BCA Protein Assay Kit (Thermo Fishcer Scientific) was used for protein quantification. Separation of proteins was performed using 12% SDS-PAGE then transferring them on to polyvinyldene difluoride (PVDF) membranes (GE10600021 Sigma, Sigma-Aldrich, MO, U.S.A.). The membranes were blocked with 5% skim milk then incubated for 1 h with the primary anti-β-actin, anti-BCL-xL, and anti-Bax monoclonal rabbit antibodies provided by Cell signaling Technology (Antibodies for β-actin: 4970S, BCL-xL: 2764T, Bax: 2772T, California, U.S.A.). The membranes then underwent incubation with secondary HRP-conjugated IgG goat anti-rabbit antibodies (7074P2, Cell Signaling Technology, Danvers, Massachusetts, U.S.A.). Using the ChemiDoc MP imaging system (Bio-Rad, Hercules, California, U.S.A.) for image processing, protein expression and quantification were carried out.

### Cell cycle analysis through flow cytometry

Flow cytometry was used to assess the cell cycle in TAMR-1 cells in response to treatment with 39.25 µg/ml DEX, 12.38 + TAM, or their combination. Cells were grown to 70% confluency in growth medium and collected after treatment for 48 h. Following trypsinization, the cells were fixed with 70% (v/v) ethanol overnight at 4°C. Cells were rinsed twice and then resuspended in PBS supplied by Beyotime (Jiangsu, China) containing 50 mg/ml PI and 0.1 mg/ml RNase A, followed by incubation for 30 min at 37°C in the dark. The cells were analyzed by a LSR II FACS flow cytometer supplied by BD Biosciences (New Jersey, U.S.A.) to identify the cell distribution in each stage of the cell cycle.

### Cell migration assay

The migration of TAMR-1 cells was examined through a wound healing assay. After seeding the cells on to 6-well plates, a pipette tip was used to create a wound across the wells. Excess cells were removed by rinsing the wells. The media was aspirated, and the wells were treated with 39.25 µg/ml of DEX, 12.38 µg/ml of TAM, or their combination, while untreated wells represented controls at 0 and 48 h. An inverted microscope (DFC290, Leica, Wetzlar, Germany) was used for image capture and migration distance was analyzed by measuring the size of the wound gap.

### SOX-2 and E2F3 gene expression determination using RT-qPCR

Total RNA was isolated from treatment and control samples using a total RNA purification kit provided by Jena Bioscience (Munich, Germany). Applied Biosystem (Foster City, California, U.S.A.) provided a cDNA archive kit for the conversion of RNA to cDNA. Using the GoTaq PCR master mix from Promega Co. (Madison, U.S.A.), quantitative PCR was carried out by mixing 25 µl of the master mix with 1 µl of cDNA, 1 µl of each primer, and 0.25 µl of CXR Reference Dye. A 7500 Real-Time PCR System from Applied Biosystems (Foster City, CA, U.S.A.) was used for the protocol, which included 40 cycles of denaturation at 95°C for 15 s and annealing for 1 min. Values were recorded as relative expression levels adjusted to GAPDH after analysis using the 2^−ΔΔCt^ technique [98]. Primer sequences, accession numbers, and melting temperatures (*T*m) are listed in Table 1.

**Table 1:** RT-qPCR forward and reverse primer sequences, accession numbers, and annealing temperatures

Ge	Accession Number	Forward Primer	Reverse Primer	Amplicon Size (bp)	*T*m
SOX-2	NM_003106.4	5′-GAGCTTTGCAGGAAGTTTGC-3′	5′-GCAAGAAGCCTCTCCTTGAA-3′	190	57.81°C
E2F3	NM_001243076.3	5′-TATCCCTAAACCCGCTTCC-3′	5′-TTCACAAACGGTCCTTCTA-3′	159	53.61°C
GAPDH	NM_001357943.2	5′-CAAAGTTGTCATGGATGACC-3′	5′-CCATGGAGAAGGCTGGGG-3′	195	54.58°C

## Supplementary Material

Supplementary Figures S1 and Table S1

## Data Availability

The data that support the findings of this study are available from the corresponding author upon request.

## Abbreviations

BC: breast cancer
CI: combination index
CSC: cancer stem cell
DEX: dexamethasone
DRI: dose reduction index
ER: estrogen receptor
E2F3: E2F transcription factor 3
GC: glucocorticoid
GR: glucocorticoid receptor
SOX-2: SRY-like box 2
SRB: sulforhodamine-B
SRY: sex determining region Y
TAM: tamoxifen
