# Supplementary material for: Dexamethasone–tamoxifen combination exerts synergistic therapeutic effects in tamoxifen-resistance breast cancer cells
Source: Biosci Rep. 2024 Jul 5;44(7):BSR20240367. doi: 10.1042/BSR20240367 (PMC11230869; doi:10.1042/BSR20240367)
Supplement: Supplementary Figures S1 and Table S1 [file BSR-2024-0367_supp.pdf]

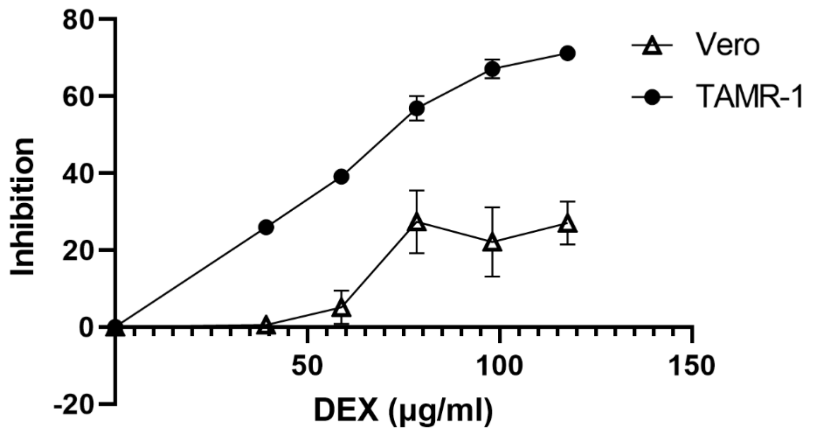

Figure S1. The cytotoxicity of DEX on human normal Vero cells in comparison with TAMR1 cells. The cytotoxicity of DEX was assessed on Vero normal cell lines. Cells were then incubated for 48 h and stained with SRB. The inhibitory effect of each drug was assessed through determining the percentage inhibition. Percentage inhibition = 100 – (100*optical density (treated cells)/optical density (untreated cells)).

Table S1. CI and DRI values of TAMR-1 cells treated with different DEX and TAM combinations in a constant 3:1 ratio.

Dose DEX (µg/mL)	Dose TAM (µg/mL)	Effect	CI	DRI DEX	DRI TAM
39.25	12.38	0.83973	0.44295	4.55902	4.47209
58.87	12.38	0.86007	0.50195	3.34277	4.93095
78.49	12.38	0.84427	0.64869	2.32699	4.56722
98.12	12.38	0.84747	0.74536	1.88823	4.63474
117.74	12.38	0.8536	0.82693	1.61955	4.77391
39.25	18.58	0.7718	0.72148	3.52171	2.28558
58.87	18.58	0.7176	1.02273	1.98686	1.92521
78.49	18.58	0.75313	1.06887	1.65764	2.14777
98.12	18.58	0.787	1.08898	1.48349	2.41024
117.74	18.58	0.77007	1.28397	1.17884	2.29526
39.25	24.77	0.79113	0.81058	3.76327	1.83534
58.87	24.77	0.83573	0.79039	2.98661	2.19511
78.49	24.77	0.8488	0.84999	2.37514	2.33123
98.12	24.77	0.8198	1.08376	1.67730	2.05101
117.74	24.77	0.78073	1.39231	1.20966	1.76794
39.25	30.96	0.79733	0.92555	3.84775	1.50227
58.87	30.96	0.76827	1.16898	2.32029	1.35501
78.49	30.96	0.78007	1.26102	1.81039	1.41112
98.12	30.96	0.78553	1.37251	1.47595	1.43890
117.74	30.96	0.79667	1.44826	1.28008	1.49912
39.25	37.15	0.778	1.13450	3.59550	1.16771
58.87	37.15	0.72033	1.52979	2.00228	0.97054
78.49	37.15	0.74227	1.58761	1.60279	1.03767
98.12	37.15	0.7712	1.58826	1.40561	1.14047
117.74	37.15	0.74027	1.91146	1.06194	1.03115

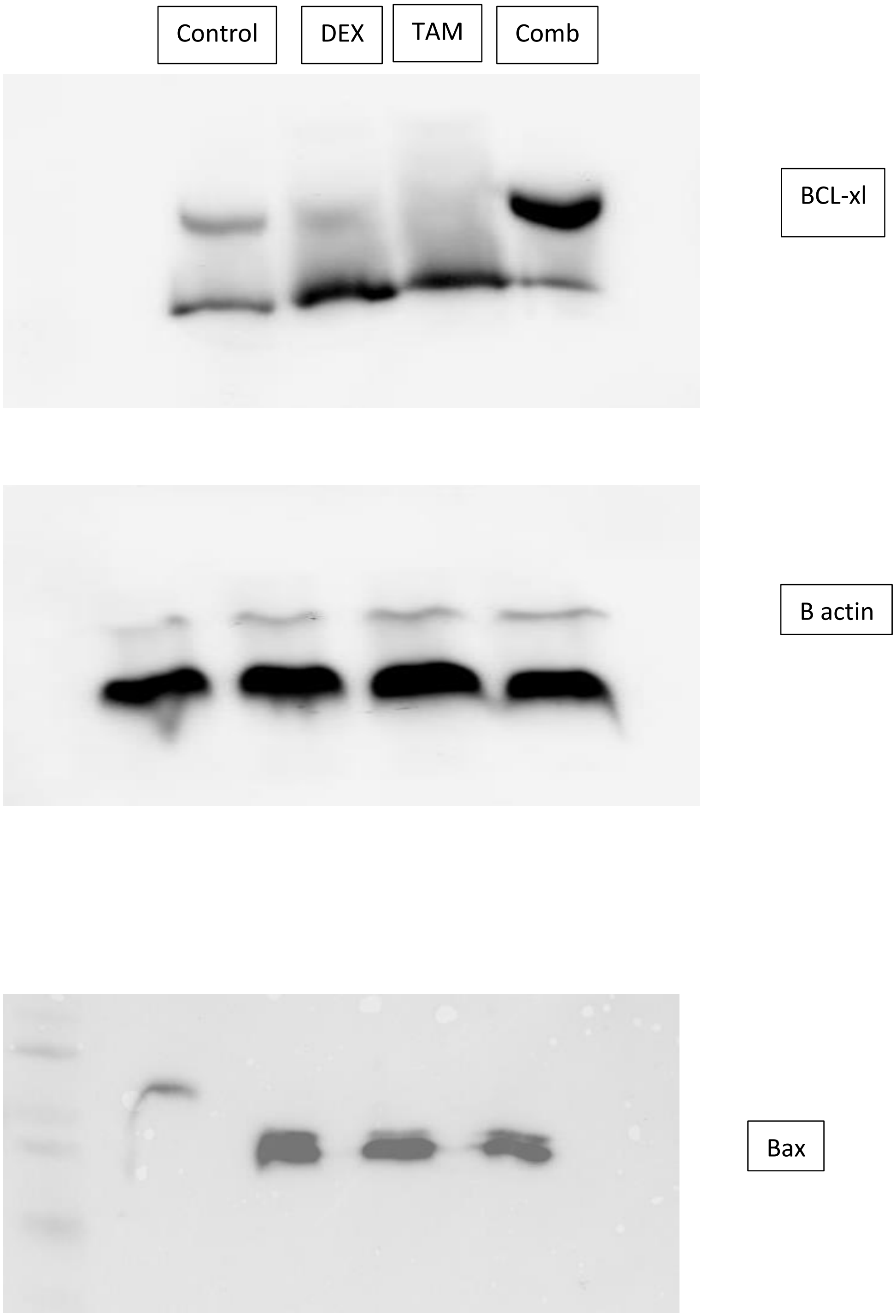
Control
DEX
TAM
Comb
BCL-xl
B actin
Bax

Control DEX TAM Comb

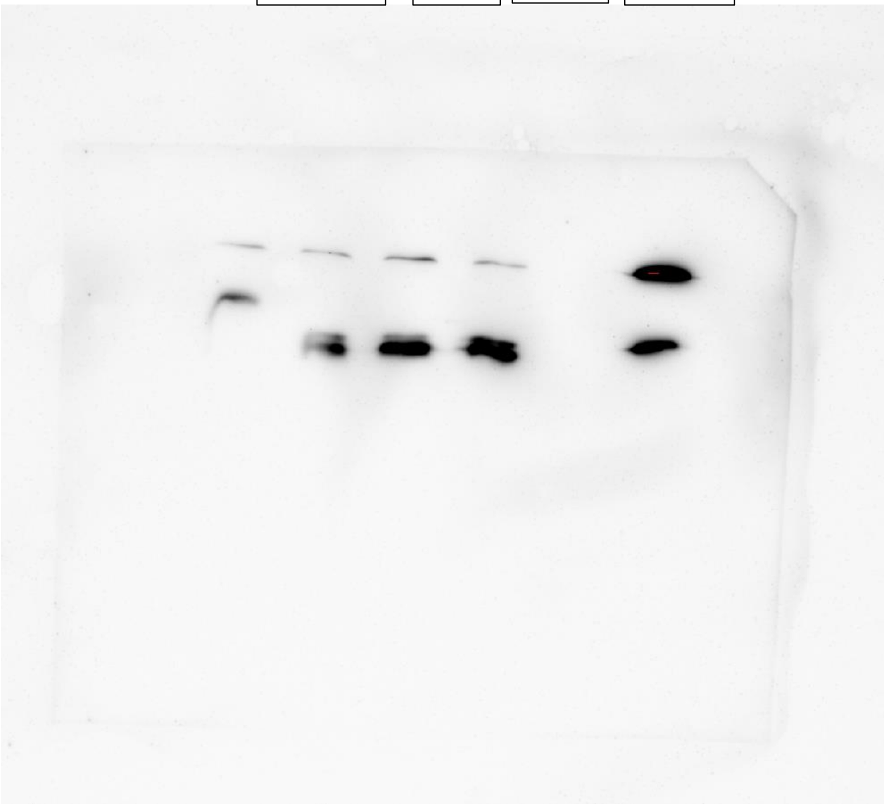

Bax, 20KDa

Control DEX TAM Comb Control DEX TAM Comb

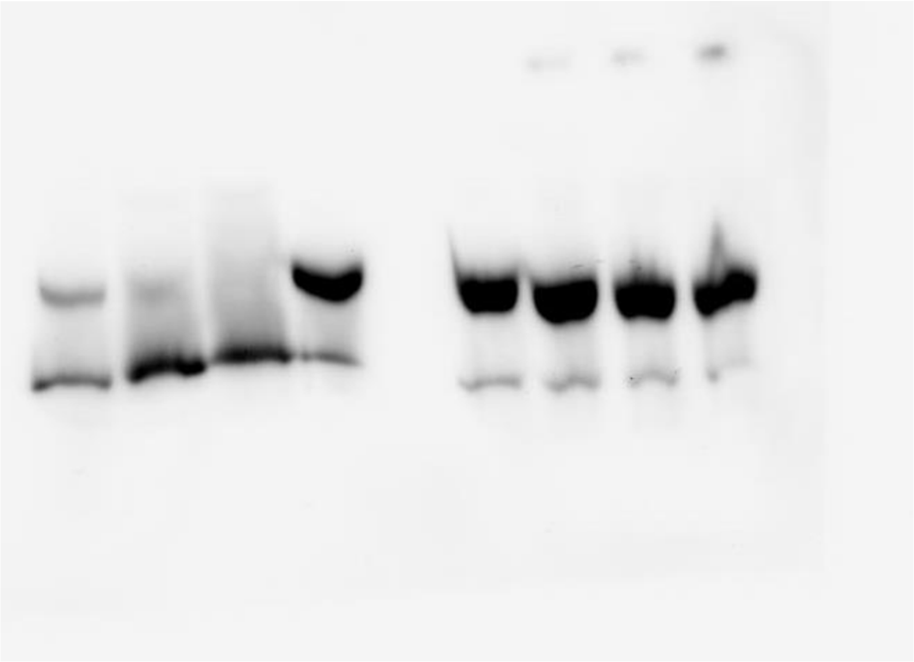

B- actin (45 KDa)

BCL-xl (30 KDa)
